# Efficacy of MEK inhibition in a recurrent malignant peripheral nerve sheath tumor

**DOI:** 10.1038/s41698-021-00145-8

**Published:** 2021-02-12

**Authors:** Sumanth Nagabushan, Loretta M. S. Lau, Paulette Barahona, Marie Wong, Alexandra Sherstyuk, Glenn M. Marshall, Vanessa Tyrrell, Eva A. Wegner, Paul G. Ekert, Mark J. Cowley, Chelsea Mayoh, Toby N. Trahair, Philip Crowe, Antoinette Anazodo, David S. Ziegler

**Affiliations:** 1 Kids Cancer Centre, Sydney Children’s Hospital, Randwick, NSW Australia; 2 School of Women’s and Children’s Health, UNSW Sydney, Sydney, NSW Australia; 3 Children’s Cancer Institute, Lowy Cancer Research Centre, UNSW Sydney, Sydney, NSW Australia; 4 Department of Nuclear Medicine and PET, Sydney Children’s Hospital and Prince of Wales Hospital, Randwick, NSW Australia; 5 Prince of Wales Clinical School, UNSW Sydney, Sydney, NSW Australia; 6 Nelune Comprehensive Cancer Centre, Prince of Wales Hospital, Randwick, NSW Australia; 7 Sydney Sarcoma Unit, UNSW Sydney, Sydney, NSW Australia

**Keywords:** Paediatric cancer, Computational biology and bioinformatics, Molecular medicine

## Abstract

The prognosis of recurrent malignant peripheral nerve sheath tumors (MPNST) is dismal, with surgical resection being the only definitive salvage therapy. Treatment with chemoradiation approaches has not significantly improved patient outcomes. Similarly, trials of therapies targeting MPNST genomic drivers have thus far been unsuccessful. Improved understanding of the molecular pathogenesis of MPNST indicates frequent activation of the mitogen-activated protein kinase (MAPK) cell signaling pathway. MEK inhibitors have shown activity in preclinical studies; however, their clinical efficacy has not been reported to date. We describe here a case of sustained complete response to MEK inhibition in an adolescent patient with a recurrent metastatic MPNST with multiple alterations in the MAPK pathway, guided by a precision oncology approach.

## Introduction

Malignant peripheral nerve sheath tumor (MPNST) is a rare, aggressive soft-tissue sarcoma with poor overall survival of <50%^1^. The only definitive therapy is complete resection, with >90% survival rate for patients who achieve gross total resection with clear margins^1^. In comparison, patients with unresectable or metastatic tumors have a survival rate of only 25%^1,2^. While neoadjuvant chemotherapy and focal radiation therapy are used, neither have a significant impact on patient outcomes. An improved understanding of the molecular drivers of MPNST may lead to new avenues of therapy and precision medicine approaches. Importantly, MPNST is associated with a germline alteration of neurofibromatosis type 1 (*NF1*) in 40–50% of cases^3^, with frequent somatic aberrations in the mitogen-activated protein kinase (MAPK) pathway. Foremost are recurrent mutations in *NF1* as well as other drivers, including *TP53*, *EED*, *SUZ12,* and *CDKN2A*^4,5^. Preclinical studies have shown MPNST dependence on the MAPK pathway^6^, but clinical efficacy with inhibition of MAPK kinase (MAP2K or MEK) has not been reported to date. We describe here a clinical case of successful treatment of an MPNST with a MEK inhibitor (MEKi). Our patient had a recurrent, metastatic MPNST and was enrolled on a precision medicine trial that identified multiple aberrations in the MAPK pathway. She was treated with the MEKi trametinib, with a sustained complete response of >15 months. To our knowledge, this represents evidence of a potentially effective systemic targeted therapy for MPNST.

## Results

A previously well 14-year-old female, with no history or features of NF1, presented to a peripheral hospital with a painless, rapidly growing 10-cm left breast mass. The mass was incompletely removed. Histological examination demonstrated a highly cellular tumor with closely packed spindle cells with moderate nuclear pleomorphism, hyperchromatic nucleoli, frequent mitoses (>10 per high-power field), and multifocal necrosis but no epithelial component. Immunohistochemistry revealed diffusely positive CD99, CD56, and TLE1, and moderate FLI1. The tumor equivocally stained for MDM2 and CDK4 and negatively for S100, with loss of H3K27me3. Ki67 ranged between 30 and 50%. Fluorescent in situ hybridization (FISH) analysis for SS18 and EWSR1 rearrangements was negative. The overall features were consistent with the diagnosis of a high-grade MPNST. She was transferred to our unit for further care. Staging with F18-fluorodeoxyglucose positron emission tomography/computerized tomography (FDG-PET/CT) confirmed localized disease. She underwent a skin-sparing mastectomy and implant reconstruction with a complete microscopic surgical clearance. She subsequently underwent fertility preservation with an egg harvest, followed by 5550 cGy of focal radiation in 30 fractions and four cycles of ifosfamide/doxorubicin chemotherapy. Post-treatment surveillance was performed using CT/MRI (magnetic resonance imaging) and FDG-PET/CT. She had a clinically detected 35-mm local recurrence within the surgical and radiation field, 12 months from diagnosis, 4 months after completion of multimodal therapy, and underwent extensive surgical clearance, including chest wall excision with clear surgical margins. Histological examination demonstrated spindled cells, high-grade atypia, hyperchromatic nuclei with frequent mitoses up to 60 per 10 high-power field, and absence of glandular or squamoid differentiation, with Ki67 ranged between 30 and 80%. The overall features were consistent with the diagnosis of a high-grade MPNST and were identical to the original diagnostic specimen. The relapse sample was submitted for comprehensive genomic profiling on the Australian **PR**ec**IS**ion **M**edicine for Children (PRISM, NCT03336931) trial, to identify potential targetable molecular aberrations^7^.

**Fig. 1:**
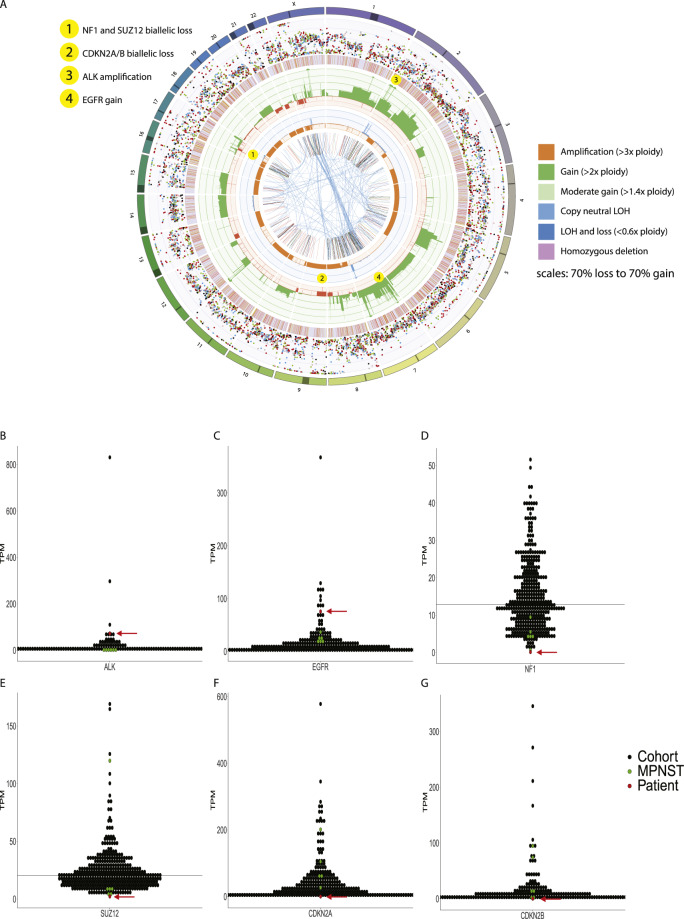
Results of genomic profiling. **A** Circos plot illustrating complex genomic profile. Outermost circle: chromosomes; Second circle: purity-adjusted allelic frequency of all observed somatic SNV, colored according to its Cosmic signature (http://cancer.sanger.ac.uk/cosmic/signatures); Third circle: small (<50 bp) insertions (yellow) and deletions; Fourth circle: copy number changes, including deletions^45^ and amplifications. If the absolute copy number is >6, it is shown as 6 with a green dot; the fifth circle: minor allele copy number, where the loss of heterozygosity is shown in orange and amplification of the minor allele shown in blue. Innermost circle: structural variants with translocations in blue, deletions in red, insertions in yellow, tandem duplications in green, and inversions in black. **B**–**G** RNA abundance was assessed for *ALK, EGFR, NF1, SUZ12, CDKN2A,* and *CDKN2B* using RNAseq and represented as transcripts per million (TPM). The red dot and arrow represent this case, relative to *n* = 5 unrelated MPNST tumors and *n* = 133 high-risk pediatric tumors from the PRISM cohort (black).

Whole-genome (paired tumor and germline) sequencing, transcriptomic (tumor) sequencing, and DNA methylation analysis was performed. The analyses demonstrated tumor purity of 98% and low tumor mutation burden (3 mutations/megabase). A somatic pathogenic *TP53* p.R273H mutation with copy-neutral loss of heterozygosity was identified. The tumor demonstrated a complex genomic profile with multiple structural and copy number events (Fig. 1A). They included homozygous deletion of 17q11.2 resulting in biallelic deletion of NF1 and *SUZ12* with low RNA expression (Fig. 1B–D), biallelic loss of *CDKN2A/2B* with low RNA expression, *ALK* amplification (ten copies) with high RNA expression, and *EGFR* copy gain (four copies) with high RNA expression (Fig. 1E, F). DNA methylation (Illumina Infinium Human Methylation 450 Array) analysis performed using the DKFZ sarcoma classifier (https://www.molecularneuropathology.org/mnp/classifier/3) classified the tumor as an MPNST (score 0.99, German Cancer Research Center DKFZ). Neither *NF1* nor other known cancer predisposition germline mutations were identified.

**Fig. 2:**
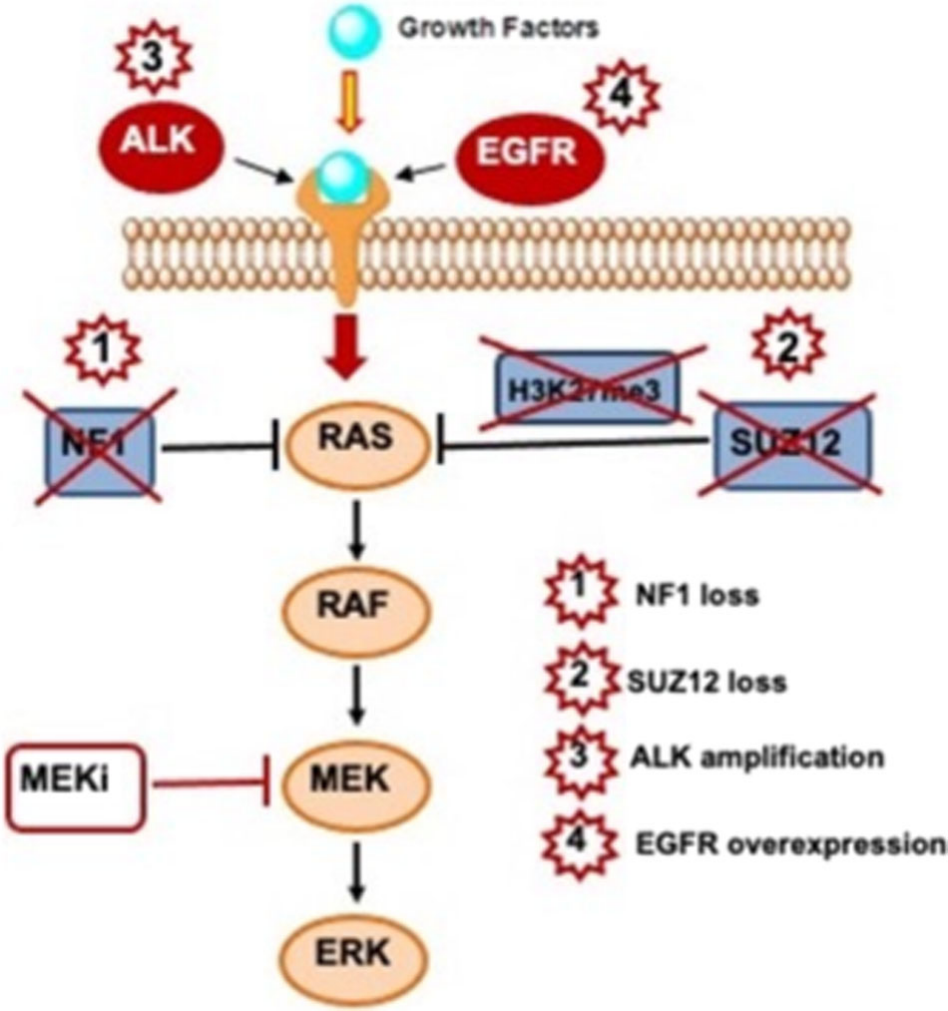
Schematic of somatic molecular aberrations. WGS and transcriptomic sequencing demonstrated multiple aberrations in the MAPK pathway, supporting downstream targeting with a MEK inhibitor (MEKi).

The tumor harbored multiple aberrations in the MAPK pathway (Fig. 2). *NF1* encodes neurofibromin 1, a RAS–GTPase-activating protein. Its negative regulatory function is attributed to a central GAP-related domain (GRD) region that is similar to ras–guanosine-triphosphate (GTP)ase activation proteins (GAPs). GAPs inactivate RAS by accelerating the conversion of active Ras–GTP to its inactive guanosine diphosphate (GDP)-bound form, preventing downstream pathway activation^8^. Hence, biallelic deletion of *NF1* leads to loss of NF1 function and increased RAS signaling^9,10^. SUZ12, a core component of the PRC2 complex, is essential to epigenetic regulation^11^. SUZ12 loss potentiates the effects of NF1 loss by amplifying downstream RAS activity through the loss of H3K27me3 and aberrant transcriptional activation^4,12^.

ALK activation can induce cellular differentiation through the MAPK pathway^13^ but could conversely confer resistance to ALK inhibitor monotherapy^14^. EGFR activation is known to induce MAPK activation through G-protein-coupled receptor kinase 2 (GPRK2)^15^. While the biallelic loss of NF1 is a well-established activating event in syndromic NF1 and its associated malignancies like glioma, leukemia, plexiform neurofibroma, MPNST, and melanoma^16–18^, the impact of ALK amplification, low EGFR copy number gain, and SUZ12 biallelic deletion on both MAPK pathway activation and sensitivity to MEK inhibitors is less certain.

Biallelic loss of CDKN2A (p16-INK4A) results in p16-mediated cell cycle promotion through the cyclin D-CDK4/6-pRb phosphorylation route^19^. This tumor carried wild-type *RB1*, which is essential for CDK4/6 inhibitor sensitivity^20^. Co-inactivation of p16 and TP53 has been observed in ~75% of MPNSTs, leading to functional consequences on cell growth control and apoptosis, with potential impact on CDK4/6 inhibitor efficacy^21^.

**Fig. 3:**
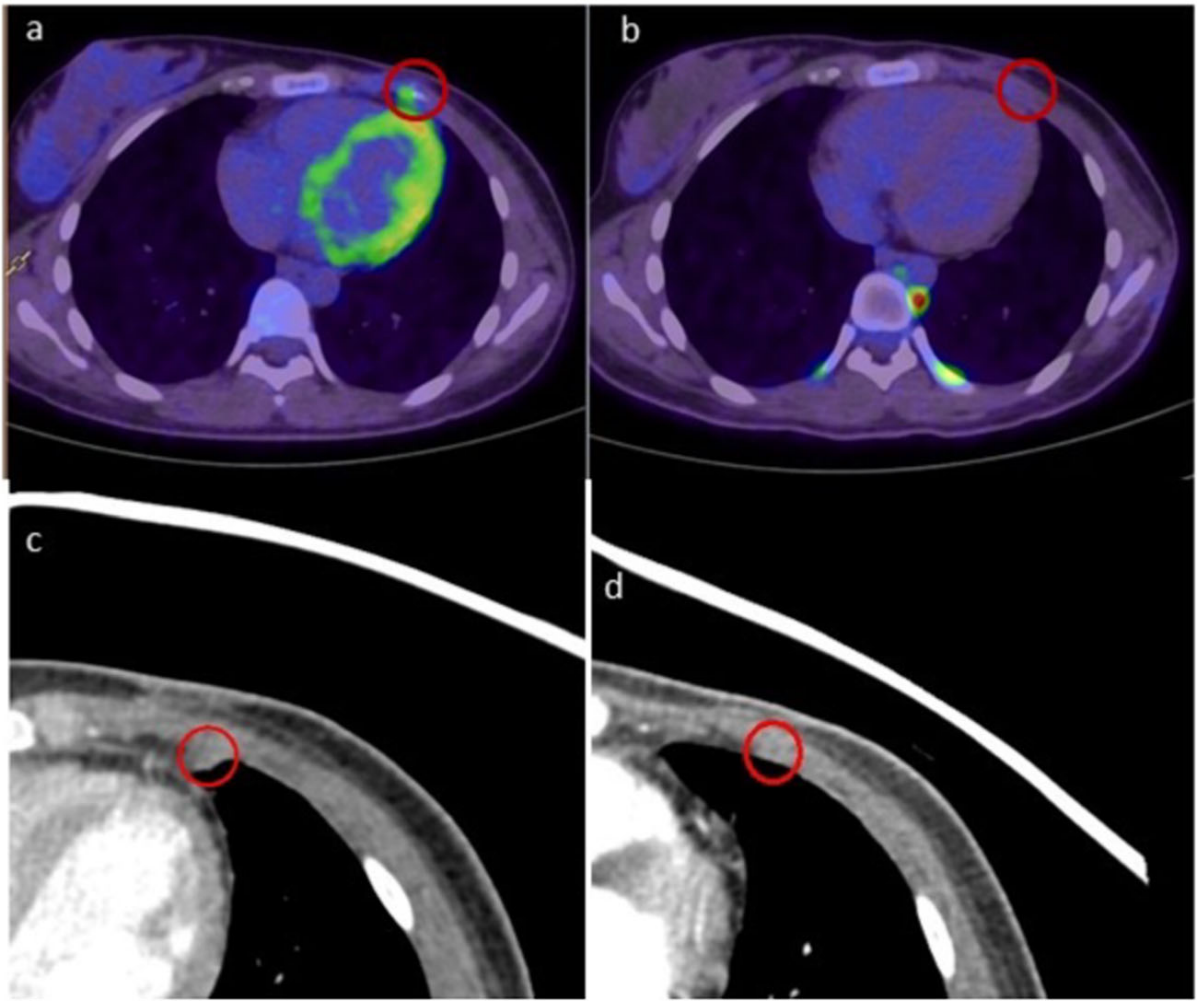
Comparative fused FDG-PET/CT images of the left 4th intercostal space nodule. **a** Focal avid disease (red circle) before commencing trametinib in May 2019. **b** Complete metabolic response after 15 months of treatment in September 2020 (with physiological brown fat activity in paravertebral and costovertebral regions). Comparative high-resolution CT images demonstrating (**c**) 6.5-mm lesion before commencing trametinib and (**d**) complete radiological response after 6 months of treatment with trametinib in October 2019.

Following the second resection, the patient underwent 6 months of radiological surveillance but experienced a second metastatic relapse in the left 4th anterior intercostal region, adjacent to the previous chest wall resection. This 6.5-mm lesion was not biopsied and treatment was guided by the genomic profiling of the first relapse sample. Given the multiple MAPK pathway aberrations, she was commenced on trametinib 2 mg daily, achieving a complete remission of the metastatic recurrence in 3 months. Serial disease response assessments by CT and FDG-PET/CT imaging showed complete resolution of the metastatic lesion (Fig. 3) with an ongoing complete metabolic response more than 15 months after the second relapse. There has been no recurrence of the primary chest lesion since the second surgery or at any other site. The only side effect of the treatment was Grade 2 acneiform rash.

## Discussion

MPNST is an aggressive nonrhabdomyomatous soft-tissue sarcoma arising from perineural and Schwann cells of the peripheral nerve sheath, with a high rate of recurrence and metastasis. They occur sporadically or in association with a germline alteration of NF1 in ~50% of cases^22^. Patients with *NF1* germline mutations carry ~10% lifetime risk of developing MPNST^3^. While resectable tumors with clear margins carry good outcomes, survival for all other patients remains poor, with the majority ultimately succumbing to the disease^1,2^. The prognosis for relapsed pediatric MPNST is exceedingly poor, with a median survival of 11 months and 5-year overall survival of 15%^23^. Relapsed tumors are insensitive to cytotoxic therapies and second-line treatments other than aggressive surgery have limited value in improving outcomes^23^. There is thus an urgent need for novel therapeutic approaches.

Trials of targeted agents have all thus far failed in MPNST, including targeted monotherapy with erlotinib, sorafenib, imatinib, dasatinib, and alisertib^24–27^. Similarly, combination trials of everolimus and bevacizumab or ganetespib and sirolimus have not shown clinical activity in recurrent MPNST^28,29^.

The genomic landscape of MPNST includes recurrent alterations in *NF1* (87.5%), *CDKN2A* (75%), *SUZ12* (56%), *TP53* (40%), and *EED* (32.5%)^5^. The striking feature of multilevel MAPK pathway activation makes clinical translation with MAPK-targeted agents attractive. The range of somatic aberrations identified in this case offered multiple potential targets and targeted therapy options. EGFR inhibition with erlotinib has shown promising preclinical anti-MPNST activity but lacked clinical efficacy in adults with metastatic or unresectable MPNST^24,30,31^. There is no current preclinical or clinical data for ALK monotherapy activity in pediatric MPNST and EGFR and ALK targeting could potentially encounter therapy resistance due to *NF1* and *SUZ12* loss-of- function mutations^14^.

In this *RB1* wild-type tumor, the biallelic loss of *CDKN2A* was potentially targetable with CDK4/6 inhibitors. Anti-tumor activity has been observed in preclinical models of RB1-preserved MPNST when treated with palbociclib^20,32^. However, CDK4/6 inhibitors are generally inactive clinically as single agents.

In contrast, MAPK signaling suppression has been shown in in vitro MPNST models with MEK inhibitors such as trametinib and selumetinib^33^. Trametinib treatment resulted in tumor growth reduction in murine models of NF1-null MPNST^34^. Mirdametinib (PD0325901), a highly selective MEK inhibitor, demonstrated a reduction in cell proliferation in vitro and prolonged mice survival in in vivo NF1-negative MPNST models^6^. Clinical efficacy of MEK inhibitor monotherapy in MPNST has not been reported to date, but a Phase 2 trial is testing the combination of selumetinib and sirolimus (mTOR inhibitor) in MPNST (NCT03433183). Selumetinib has demonstrated clinical activity with objective responses in 71% of children with NF1-driven plexiform neurofibromas in Phase 1 clinical trial^9^. A recent Phase 2 trial in children with NF1-driven plexiform neurofibromas showed 70% partial tumor response with meaningful improvement in pain and quality-of-life parameters with selumetinib^35^. Similarly, trametinib has shown a reduction in tumor volume in recurrent pediatric low-grade gliomas and NF1-driven plexiform neurofibromas^36–38^.

Therapeutic decision-making was challenging in this case, but factors favoring the choice of trametinib were the biological rationale, its ability to target downstream molecular abnormalities and alleviate therapy resistance, accessibility in Australia outside a clinical trial setting, and experience in monitoring and managing drug toxicity. The patient has tolerated trametinib well, apart from moderate acne, which is improving on cessation of the drug and has remained in continuous complete remission for more than 15 months. To our knowledge, objective response to a MEK inhibitor in relapsed MPNST has not been described in the literature to date. We provide evidence of the utility of a personalized approach for these rare, aggressive tumors, minimizing off-target toxicity, and positively impacting quality of life.

It is important to note that there were some unusual clinical features in this case. MPNST is uncommon in childhood and cancer is more often seen in young and middle-aged adults, accounting for 5% of soft-tissue sarcomas in patients less than 20 years of age^18^. Although primary MPNSTs of the breast have been sporadically reported in adults^39,40^, they are rare in the pediatric age group^41^, particularly in the absence of any clinical or germline features of NF1. Moreover, high-grade MPNSTs are usually associated with distant than local metastatic recurrence^42^. The genomic changes, however, are consistent with those described in both NF1- associated and sporadic cases of MPNST. H3K27 trimethylation loss is associated with high-grade MPNSTs and correlates with poor survival^43,44^. This patient’s disease has shown an objective response in the presence of these unusual and high-risk features. The clinical response seen in this patient needs to be confirmed in other patients, ideally through evaluation in a clinical trial, to determine whether the results can be replicated.

Improving our understanding of the molecular basis of MPNST through genomic, transcriptomic, drug screening responses, and tumor microenvironment approaches is critical to the discovery of new effective therapy for this challenging disease. Given the high proportion of MPNST tumors with aberrations in the MAPK pathway, treatment with MEK inhibitors may represent an effective therapy for many patients with the appropriate genotype and merits further prospective evaluation through clinical trials.

## Methods

### Whole-genome sequencing

DNA was extracted from tumor: normal matched-pair tissue and libraries were prepared using KAPA Hyper PCR-Free Library Preparation Kit (Roche Inc). Both normal and tumor libraries were sequenced on Illumina HiSeq X Ten to generate 2 × 150-bp reads at a target coverage of >30× for germline and >90× for tumor samples. WGS was conducted at the Kinghorn Centre for Clinical Genomics, Garvan Institute of Medical Research (Australia). WGS data analysis was conducted as previously described^7^. Raw fastq files were aligned to the hs37d5 reference genome using BWA-MEM (v0.17.10-r789), with the resulting BAM files merged and duplicate reads marked using Novosort (v1.03.01, default settings), and read alignment improved using GATK Indel Realignment (v3.3). Germline SNVs and short (<50-bp) indels were identified using GATK HaplotypeCaller, GenotypeVCFs, and VQSR (all v3.3), annotated with VEP (v87), converted into a GEMINI (v0.11.0) database, and imported into Seave for filtration and prioritization. Somatic SNVs and short indels (<50 bp) were identified using Strelka (v2.0.17). Somatic variants were annotated using SnpEff (v4_3t). Tumor purity, ploidy, and somatic copy number alteration were identified using PURPLE (v2.39), and somatic SVs were identified using GRIDSS (v2.7.2) and then annotated using Ensembl genes. Somatic and germline variant curation was conducted as described^7^.

### RNA sequencing

RNAseq was conducted at the Murdoch Children’s Research Institute (Australia) and performed with the TruSeq Stranded mRNA Preparation Kit. Libraries were pooled, and sequencing runs were performed in paired-end mode using the NextSeq 500 platform generating at least 80 million reads. RNA data analysis was performed as previously described^7^. Initial RNAseq quality control (QC) checks included an evaluation of GC and per-base sequence content using FastQC (v0.11.5). Reads were aligned to the human genome assembly (build hg19) using the STAR (v2.5) two-pass method with quantMode parameters set to TranscriptomeSAM for alignments translated into transcript coordinates. Alignments were sorted with SAMTools (v1.3.1), duplicates were marked with Picard Tools (v2.4.1), reads were split and trimmed, and mapping qualities were reassigned with the Genome Analysis Toolkit (v3.6) using the methods SplitNCigarReads and ReassignOneMappingQuality, respectively. Post-alignment QC required at least 70% of reads to be uniquely aligned, assessed using STAR alignment statistics (100% of samples passed). Raw gene counts and transcripts per kilobase million (TPM) were calculated using RSEM (v1.2.31). All RNAseq expression values are represented as TPM. Variants were called on RNA with GATK HaplotypeCaller (v3.6), and ANNOVAR (v20190929) was used for variant annotation. Fusions were identified using three methods: STAR-Fusion (v1.3.1), JAFFA (v1.09), and Arriba (v1.1.0, https://github.com/suhrig/arriba/).

### DNA methylation

DNA methylation analysis was conducted at the Australian Genome Research Facility (Australia). Tumor DNA was extracted followed by bisulfite conversion using Zyma EZ DNA Methylation kit. Samples were hybridized on the Illumina 850 K Infinium Methylation EPIC BeadChip. Methylation data were uploaded into the DKFZ methylation analysis portal for sarcoma classification (https://www.molecularneuropathology.org/mnp/classifier/3).

### Ethics approval

Ethical approval for the PRISM trial was granted by the Hunter New England Human Research Ethics Committee, New South Wales, Australia, in accordance with the National Health and Medical Research Council’s National Statement on Ethical Conduct in Human Research (2007) (National Statement) and the CPMP/ICH Note for Guidance on Good Clinical Practice (Ref: HREC/17/HNE/29). Written informed consent was provided by the parents prior to enrolment on the PRISM trial.

### Reporting summary

Further information on research design is available in the Nature Research Reporting Summary linked to this article.

## Supplementary information

nr reporting summary

## Data Availability

The data generated and analyzed during this study are described in the following data record: 10.6084/m9.figshare.13469001^45^. Whole-genome sequencing, RNA sequencing, and DNA methylation data are available from the European Genome-phenome Archive under dataset accession https://identifiers.org/ega.dataset:EGAD00001006793 (study accession: https://identifiers.org/ega.study:EGAS00001004899)^46^. The data are controlled access but are publicly available after Data Access Agreement consent.

## Code availability

Software and scripts related to this publication are available at https://github.com/CCICB/2020-hrPC-landscape.
